# Assessing Clinical Change in Individuals Exposed to Repetitive Head Impacts: The Repetitive Head Impact Composite Index

**DOI:** 10.3389/fneur.2021.605318

**Published:** 2021-07-06

**Authors:** Charles Bernick, Guogen Shan, Lauren Bennett, Jay Alberts, Jeffrey Cummings

**Affiliations:** ^1^Neurological Institute, Cleveland Clinic, Las Vegas, NV, United States; ^2^Department of Neurology, University of Washington, Seattle, WA, United States; ^3^Department of Epidemiology and Biostatistics, University of Nevada, Las Vegas, NV, United States; ^4^Neurological Institute, Cleveland Clinic, Cleveland, OH, United States; ^5^Center for Transformative Neuroscience, University of Nevada, Las Vegas, NV, United States

**Keywords:** traumatic brain injury, chronic traumatic encephalopathy, composite index, cognition, composite index

## Abstract

**Background:** There is a current lack of any composite measure for the effective tracking and monitoring of clinical change in individuals exposed to repetitive head impacts (RHI). The aim of this study is to create a composite instrument for the purposes of detecting change over time in cognitive and behavioral function in individuals exposed to RHI.

**Methods:** The data to derive the composite instrument came from the Professional Fighters Brain Health Study (PFBHS), a longitudinal study of active and retired professional fighters [boxers and mixed martial arts (MMA) fighters] and healthy controls. Participants in the PFBHS underwent assessment on an annual basis that included computerized cognitive testing and behavioral questionnaires. Multivariate logistic regression models were employed to compare active fighters (*n* = 117) with controls (*n* = 22), and retired fighters (*n* = 26) with controls to identify the predictors that could be used to differentiate the groups over time. In a second step, linear discriminant analysis was performed to derive the linear discriminant coefficients for the three groups by using the predictors from the two separate logistic regression models.

**Results:** The composite scale is a weighted linear value of 12 standardized scores consisting of both current and yearly change scores in domains including: processing speed, choice reaction time, semantic fluency, letter fluency, and Barrett Impulsiveness Scale. Because the weighting of values differed between active and retired fighters, two versions emerged. The mean and standard deviation ratio (MSDR) showed that the new index had better sensitivity compared to the individual measures, with the ratio of MSDR of the new index to that of the existing measures of at least 1.84.

**Conclusion:** With the increasing need for tools to follow individuals exposed to RHI and the potential of clinical trials on the horizon for CTE, the RHICI is poised to serve as an initial approach to a composite clinical measure.

## Introduction

There is increasing interest in understanding the long term neurological effects of exposure to repetitive head impacts (RHI) including Chronic Traumatic Encephalopathy (CTE), with the anticipation of developing therapeutic interventions (1–3). The tools we apply to identify cognitive and behavioral changes in those exposed to RHI are borrowed from standard neuropsychological tests and behavioral instruments. While casting a broad net by utilizing an array of discrete tests may capture the range of deficits one might detect in those exposed to RHI, it would be helpful to have a single measure that can be used to track change clinically and in epidemiological studies and clinical trials. Currently, though, we lack any composite measure that is focused on the domains that are most likely to show change due to RHI.

To develop and validate new test batteries takes significant time and effort. Composite scales can be produced by either consensus (e.g., United Parkinson's Disease Research Scale) or data driven [e.g., Alzheimer's disease Composite Score (ADCOMS)] strategies (4–6). The data-driven approach leverages existing scales and uses mathematical approaches to identify the items most sensitive to change in the group being studied and applies weighting to improve performance.

Previous work on neuropsychological and behavioral changes in those exposed to RHI have tended to be cross sectional and have reported deficits in domains such as memory, information-processing speed, finger-tapping speed, complex attentional tasks, and frontal-executive functions (7–9). However, our experience from the Professional Fighters Brain Health Study (PFBHS), a longitudinal study of professional boxers and mixed martial arts (MMA) fighters, has suggested that not all cognitive domains are affected equally, particularly when viewed on a longitudinal basis (10–13). Moreover, it has been reported that behavioral changes can be a prominent feature in those exposed to RHI, particularly aspects of impulsivity and behavioral dyscontrol (14, 15).

The aim of this study is to create a composite instrument that could detect change over time in clinical measures in individuals exposed to RHI. This was accomplished by utilizing data obtained from the Professional Fighters Brain Health Study (16). The composite scale—termed Repetitive Head Impact Composite Index (RHICI)—if validated in prospective studies, could be used for natural history studies of RHI, clinical trials of traumatic encephalopathy syndrome (TES), or other mild traumatic brain injury research.

## Methods

The data used for the RHICI came from the PFBHS, a convenience sample consisting of active and retired professional fighters (boxers and MMA fighters) and healthy controls. Active fighters were required to have at least 1 professional fight within 2 years of enrollment and be training with the intent to compete. Retired fighters were included if they had been boxers, had a minimum of 10 professional fights, had no sanctioned fights for at least 2 years, and did not intend to return to competition (there were too few retired MMA fighters to include as a separate group). Control subjects were recruited from outreach efforts in the community and could not have any self-reported prior history of neurological disorders, head trauma, military service, or participation at a high school level or higher in a combat sport or a sport in which head impacts can be anticipated to occur, such as American football, wrestling, hockey, rugby, soccer, or rodeo. All participants were required to be able to read at a minimum at a 4th grade level but were not otherwise screened for cognitive status or subjective complaints prior to enrollment.

Enrollment in the PFBHS began in 2011 and has been continuous since. Each participant is seen on an annual basis, and for active fighters, not sooner than 45 days after a sanctioned fight. The PFBHS was approved by the Cleveland Clinic Institutional Review Board and written informed consent was obtained from all participants. More detailed methods of recruitment and study procedures have been described previously (16).

At baseline and each annual visit, a battery of tests and information were acquired including magnetic resonance imaging (MRI) of the brain, computerized cognitive testing, behavioral questionnaires, and exposure history. Participants answered questionnaires with the assistance of the study coordinator that collected information on demographics; educational attainment; medical history including concurrent illnesses and prescribed medications; previous head trauma, both related and unrelated to athletic activities; and prior involvement in other contacts sports.

Cognitive function was assessed by two computerized cognitive test batteries and verbally administered measures of verbal fluency. One computerized battery consists of four subtests of the CNS Vital Signs™ (CNS Vital Signs™, North Carolina) including verbal memory, symbol digit coding, Stroop and a finger tapping test. CNS Vital Signs™ offers robust and reliable measurements of cognition, which are computerized; test performance is supervised by a technician (17). Results from these tests are used to create scores in the following clinical domains: verbal memory, processing speed, psychomotor speed and reaction time. The other computerized cognitive assessment, C3 Logix, an iPad-based test that includes a processing speed test, Trail Making Test Parts A and B, simple and choice reaction time paradigms along with a balance measure (18). Tests of verbal fluency include both letter (words that start with “f”) and semantic (animals) fluency tasks.

Behavioral assessment was obtained through administration of the Patient Health Questionnaire (PHQ 9) that evaluates the presence of depressive symptoms and the Barrett Impulsiveness Scale (BIS II) (19, 20). The BIS II comprises 30 questions, which have been shown to load on six factors (attention, cognitive instability, motor, perseverance, self-control, and cognitive complexity); the items loading on these factors provide the subscales for the instrument. For both total and subscale scores, higher scores refer to higher levels of impulsiveness.

### Statistical Methods

#### Samples

Data from the PFBHS study was used to develop a new composite index for individuals exposed to RHI. We included participants having at least three study visits over a minimum of 2 years. The normal controls (*n* = 22) were used as the reference group with the assumption that they were the least likely to show decline in cognitive measures over a several year period. The conceptual framework informing the index was to identify and create a composite from those cognitive and behavioral measures that were able to best differentiate over time the active fighters (*n* = 117) from the controls and the retired fighters (*n* = 26) from the controls.

The initial step was to examine the correlation of the cognitive and behavioral variables with each other in a correlation matrix. This was performed to avoid the problem that could be caused by multiple co-linearity—when two highly correlated variables are both included in a statistical model may result in the sign change of parameter estimates. When two or more measures were highly correlated (*p*-value of 0.05), the study team adjudicated which one was clinically more important.

After removing the highly correlated measures, 16 items from cognitive and behavioral assessments were initially included in the new index. Among these 16 measures, 12 were cognitive measures (C) and 4 were behavioral measures (B). Although we had longitudinal data from multiple time points, the change of the last measure from baseline was used to avoid the violation of the linear assumption of the longitudinal measures. For each measure, we utilized the score and the average yearly change in score based on the last measure and the baseline, comprising a total of 32 predictors.

#### New Composite Index

Multivariate logistic regression models were employed to compare active fighters with controls, and retired fighters with controls to identify the predictors that could be used to differentiate the groups. In a second step, linear discriminant analysis was performed to derive the linear discriminant coefficients for the three groups by using the predictors from the two separate logistic regression models.

Assuming that CA and BA represent the average yearly changes of cognitive measures and behavioral measures, respectively, we standardized all the measures by using the range of each measure from all samples. The multinomial logistic regression between the active fighter group and the control group was presented as:

$$\begin{array}{l} \text{g}(\text{Pr}(\text{Y}=\text{j}|\text{X}))=\sum_{i=1}^{12}\beta_{i}C_{i}+\sum_{i=1}^{12}\gamma_{i}CA_{i}+\sum_{i=1}^{4}\varphi_{i}B_{i}\\ \;+\sum_{i=1}^{4}\omega_{i}BA_{i} \end{array}$$

where g(p) = log[p/(1–p)] is the logit link function, X are the observed data, and Y is the group with 0 for the control group and 1 for the active fighter group. A similar logistic regression model was used for the retired fighter group using the control group as the reference group. We utilized the backward model selection method with the *p*-value of 0.3 as the threshold to select the measures that can be used in the next step in developing a new composite index. The selected predictors that did not meet the direction were removed from the final model. The final model had a total of 12 measures among the 32 predictors from cognitive and behavioral assessments.

In the final step of composite construction, we performed discriminant analysis in which the group variable (control, active fighters, and retired fighters) was used as the outcome, and the 12 measures were included in the model as independent variables. Discriminant analysis was performed to derive the linear discriminant coefficients for the 12 measures in each group. We then rescaled the new index to a range of 0–20 with lower scores considered to be normal.

## Results

The characteristics of the participants that make up the data set are shown in Table 1. Retired fighters as expected were older and had more number of fights and years of fighting than active fighters, whereas the controls tended to have a slightly higher level of education than the active fighters (see Supplementary Material). The proposed composite index is a weighted linear value of the 12 standardized scores with 6 sub-scores: processing speed, choice reaction time, semantic fluency (both total correct score and number of repeated words), letter fluency (number of repeated words), and Barrett Impulsiveness Scale Question 2 (I do things without thinking); and 6 yearly change sub-scores that capture both the baseline difference and the longitudinal change over time: choice reaction time, semantic fluency (total correct and number of repeats), letter fluency (number of repeats), Barrett Impulsiveness Scale question 2 and 6 (I have racing thoughts). Because the weighting of values differed between active and retired fighters, two versions of the RHICI emerged.

**Table 1:** Characteristics of study participants in the Professional Fighters Brain Health Study that comprise the dataset used to develop the Repetitive Head Impact Composite Index.

	**Active fighters**	**Retired fighters**	**Control**	***p*-value**
*N*	117 (70.9%)	26 (15.8%)	22 (13.3%)	
Age	29.9 (6.3)	47.2 (10.0)	35.1 (14.7)	<0.0001
Education years	13.5 (2.0)	13.7 (2.1)	14.8 (2.8)	0.0496
Female	16 (13.7%)	1 (3.9%)	2 (9.1%)	0.3390
Number of fights	9.6 (11.3)	34.3 (16.6)	0	<0.0001
Years of fighting	4.6 (4.4)	9.7 (5.1)	0	<0.0001
Race				0.5686
African American	26 (22.2%)	3 (11.5%)	3 (13.6%)	
White	55 (47.0%)	16 (61.5%)	11 (50.0%)	
Other	36 (30.8%)	7 (26.9%)	8 (36.4%)	

The range of the new index was from 0 to 20. We evaluated the performance of the new index in the active fighters as compared to the individual measures with regards to sensitivity. The sensitivity was calculated as the mean and standard deviation ratio (MSDR) over a fixed follow-up time (e.g., 2 years). A larger MSDR value represented a larger effect size, which leads to greater sensitivity.

We computed the MSDR using the 2-year data of each fighter (e.g., the 1st and 3rd visits). The MSDR (Table 2) showed that the new index had better sensitivity compared to the individual measures, with the ratio of MSDR of the new index to that of the existing measures of at least 1.84.

**Table 2:** Comparison of the mean and standard deviation ratio between the Repetitive Head Impact Composite Index (RHICI) and individual scores on processing speed, choice reaction time, or letter fluency tests.

	**Mean and standard deviation ratio**	
	**Active fighters (ratio)**	**Retire fighters (ratio)**
RHICI	5.79 (1.00)	4.89 (1.00)
Processing speed score	2.23 (2.59)	2.38 (2.05)
Choice reaction time	1.94 (2.98)	2.66 (1.84)
Letter fluency total correct score	1.96 (2.95)	1.69 (2.90)

We showed substantial improvement in sensitivity as compared to the individual measures. The improved sensitivity of the RHICI would be expected to reduce the sample size for a clinical trial if used as an outcome measure. As an example, consider a hypothetical 2-year study to detect a 5% decline from baseline for active fighters using the existing measures, to attain 90% power at the significance level of 0.05. The required sample size based on processing speed score was 126; it was reduced to 25 when the new index was used. Similar results were observed when other measures were used in sample size calculations.

## Discussion

Assessment tools that are composed of elements of existing scales thought most likely to detect change in a particular disease are commonly used as outcome measures in clinical trials and observational studies (21). There are several advantages to using composite measures for clinical and research purposes including improved power to detect change (thus potentially necessitating fewer participants for clinical trials), avoiding arbitrary choices between several important outcomes that occur in the same disease, and serving as a common measure that is comparable between groups (22, 23).

Though there has been increasing attention to the long-term sequelae of RHI including CTE, no validated composite measure is available. The RHICI is a first attempt using data-driven methods to construct a composite scale to be used in tracking longitudinal change and as a potential outcome measure in studies related to neurological effects of RHI. The RHICI has the advantage of easy administration on a desktop or tablet computer, with most subtests performed on the device itself or the score entered by the person supervising the testing (verbal fluency).

The components that constitute the RHICI currently, including measures of processing speed, attention, executive function and impulsivity, are consistent with domains that have been reported in the literature as affected in boxers and other groups exposed to RHI (2, 7, 24, 25). However, some features described in cohorts exposed to RHI did not enter our model. Delayed memory did not emerge as a component of the RHICI despite memory complaints by many with RHI and impaired memory function being reported in individuals who had a confirmed pathological diagnosis of CTE (8, 15, 25). The lack of memory elements in the RHICI may reflect the predominance of executive dysfunction associated with RHI or the limited method for which we assessed memory function in the computerized battery that was employed in the PABHS. As we accumulate greater numbers of older retired fighters in our sample (or older former athletes in other samples tested), it is possible that this domain will enter the model and be added to the RHICI.

The structure and weighting of items in the RHICI differed between active and retired fighters. The reason for this difference is speculative. One possibility is that the underlying pathophysiologic process may differ between these two groups. In the active fighter group, RHICI may be measuring the effects of accumulating axonal injury whereas the retired fighters may include some who are harboring a neurodegenerative process due to CTE. Longitudinal MRI regional volumetric data suggest such a dichotomy (11). The practical implication is that in implementing the RHICI, the specific version used would need to be chosen based on whether the cohort is actively exposed to RHI or is an older previously exposed group.

A unique feature of the RHICI is the inclusion of both current values and rate of change from baseline in several elements. The RHICI is not intended to be used as a diagnostic test though a baseline score can be calculated to provide an anchor point from which clinical trajectory can be followed. Commonly used composite scales include measures that are thought to be characteristic of a particular condition and can differentiate patients from those without the disease. However, it is possible that the rate of change of some of these components may also be informative. By specifically assessing how certain tests change over time and integrating these elements into the RHICI, we expect this instrument to be of particular value in monitoring longitudinal change or as an outcome measure in clinical trials.

The use of composite measures as endpoints in clinical trials have been encouraged by the Food and Drug Administration (FDA) and European Medical Agency (EMA) provided that the measures are carefully designed, are relevant to existing tools for which historical evidence exists and are validated in independent prospective cohorts (26, 27). Given that the RHICI is based on data derived from commonly used neuropsychological tools, the first two requirements are met. However, the RHICI must be validated in separate samples. Unlike other neurodegenerative diseases such as Alzheimer's disease where large longitudinal datasets are publicly available, these types of data are more limited for those exposed to RHI (28). The PFBHS has over 100 additional participants who will be undergoing their third time point assessment next year; we will prospectively validate the RHICI on the larger sample.

Moving forward, it will be essential to assess the RHICI in other cohorts exposed to RHI if this tool is to have broad application. While the version we describe is derived from professional combatants, it is recognized that the clinical presentation of those exposed to RHI is heterogenous and potentially related to the type of exposure to RHI (29). For example, among those exposed to RHI from American Football there are groups that present at a younger age with mainly behavioral symptoms, those that present at an older age with primarily cognitive symptoms and a group that presents with a mixture of cognitive and behavior (30). Moreover, combat sports athletes generally will have more motoric features (e.g., ataxia, parkinsonism) than those from American Football. We would anticipate that perhaps elements from other cognitive, behavioral or motor scales may eventually be part of the RHICI as we examine this measure in other groups.

### Limitations

In developing the RHICI, we utilized all the cognitive and behavioral data collected from the PFBHS, a longitudinal observational study of active and professional fighters. However, it is important to acknowledge the limitations of this study. The extent of testing included several computerized cognitive batteries and self-administered behavioral inventories but was not exhaustive, particularly in surveying memory and behavioral dysregulation. The number of participants (particularly retired fighters and controls) who had complete data for at least three time points was limited as was the number of controls in the cohort. In regard to the latter, we would anticipate as more data become available from our cohort and others that the elements that comprise the RHICI may be modified or weighted differently. The computerized assessments used are proprietary, placing limitations on their widespread use.

For the statistical models, several assumptions are made. With multiple regression analysis and linear discriminant analysis, it is assumed that there is a linear relationship between the outcome and each independent variable. However, this assumption may not be satisfied in clinically collected data and could lead to errors in interpretation Furthermore, such models could be sensitive to outliers (though the influence of such possible outliers becomes smaller when the number of participants increases relative to the number of predictor variables). Another assumption in these two statistical models is the normality of independent variables. For the three individual measures, we tested their normality by using the Kolmogorov-Smirnov test. The normality of processing speed and that of choice reaction time were satisfied with the *p*-values above 0.05, while letter fluency total correct score fails to meet the normality with the computed *p*-value below 0.05. A mathematical function (e.g., log, arcsin) may be used to transform data to meet the normality assumption.

## Conclusions

There is an increasing need for tools to effectively track individuals exposed to RHI. With the potential of treating the consequences of RHI via clinical trials on the horizon, the proposed *Repetitive Head Impact Composite Index*, a sensitive measure to change in executive function, provides a standardized approach evaluating long-term effects of RHI.

## Data Availability Statement

The raw data supporting the conclusions of this article will be made available by the corresponding author upon reasonable request.

## Ethics Statement

The studies involving human participants were reviewed and approved by Cleveland Clinic. The patients/participants provided their written informed consent to participate in this study.

## Author Contributions

CB, GS, LB, and JC were involved in design of study, analysis and interpretation of the results, and writing of manuscript. GS performed statistical analysis. JA was involved in instrument development and interpretation of results. All authors read and approved the final manuscript.

## Conflict of Interest

CB has received research funding from the Ultimate Fighters Championship, Top Rank promotions, Haymon Boxing, and Bellator/Spike TV. JA has authored intellectual property related to the C3 Logix software. JC has provided consultation to Acadia, Actinogen, AgeneBio, Alkahest, Alzheon, Annovis, Avanir, Axsome, Biogen, Cassava, Cerecin, Cerevel, Cognoptix, Cortexyme, EIP Pharma, Eisai, Foresight, Gemvax, Green Valley, Grifols, Hisun, Karuna, MapLight, Novo Nordisk, Nutricia, Orion, Otsuka, ReMYND, Resverlogix, Roche, Samumed, Samus Therapeutics, Third Rock, Signant Health, Sunovion, Suven, and United Neuroscience pharmaceutical and assessment companies. The remaining authors declare that the research was conducted in the absence of any commercial or financial relationships that could be construed as a potential conflict of interest.

## References

[1] Asken BM Sullan MJ DeKosky ST Jaffe MS Bauer RM. Research gaps and controversies in chronic traumatic encephalopathy: a review. JAMA Neurol. (2017) 74:11256–62. 10.1001/jamaneurol.2017.2396 28975240

[2] McKee AC Stein TD Kiernan PT Alvarez VE. The neuropathology of chronic traumatic encephalopathy. Brain Pathol. (2015) 25:350–64. 10.1111/bpa.12248 25904048 PMC4526170

[3] Tharmaratnam T Iskandar M Tabobondung T Tobbia I Gopee-Ramanan P Tabobondung TA. Chronic traumatic encephalopathy in professional American football players: where are we now? Front Neurol. (2018) 9:445. 10.3389/fneur.2018.00445 29971037 PMC6018081

[4] Movement Disorder Society Task Force on Rating Scales for Parkinson's Disease. The Unified Parkinson's Disease Rating Scale: status and recommendations. Mov Disord. (2003) 18:738–50. 10.1002/mds.10473 12815652

[5] Wang J Logovinsky V Hendrix SB Stanworth SH Perdomo C Xu L. ADCOMS: a composite clinical outcome for prodromal Alzheimer's disease trials. J Neurol Neurosurg Psychiatry. (2016) 87:993–99. 10.1136/jnnp-2015-312383 27010616 PMC5013117

[6] Goetz CG Tilley BC Shaftman SR Stebbins GT Fahn S Martinez-Martin P. Movement disorder society-sponsored revision of the unified Parkinsons disease rating scale: scale presentation and clinimetric testing results. Mov Disord. (2008) 23:2129–70. 10.1002/mds.22340 19025984

[7] Mendez MF. The neuropsychiatric aspects of boxing. Int J Psychiatry Med. (1995) 25:249–62. 10.2190/CUMK-THT1-X98M-WB4C 8567192

[8] Schaffert J LoBue C Fields L Wilmoth K Didehbani N Hart J. Neuropsychological functioning in ageing retired NFL players: a critical review. Int Rev Psychiatry. (2020) 32:71–88. 10.1080/09540261.2019.1658572 31592681

[9] Pearce AJ Rist B Fraser CL Cohen A Maller JJ. Neurophysiological and cognitive impairment following repeated sports concussion injuries in retired professional rugby league players. Brain Inj. (2018) 32:498–505. 10.1080/02699052.2018.1430376 29388850

[10] Bernick C Banks SJ Shin W Obuchowski N Butler S Noback M. Repeated head trauma is associated with smaller thalamic volumes and slower processing speed: the Professional Fighters' Brain Health Study. Br J Sports Med. (2015) 49:1007–11. 10.1136/bjsports-2014-093877 25633832 PMC4518758

[11] Bernick C Shan G Zetterberg H Banks S Mishra VR Bekris L. Longitudinal change in regional brain volumes with exposure to repetitive head impacts. Neurology. (2020) 94:e232–e40. 10.1212/WNL.0000000000008817 31871218 PMC7108810

[12] Mishra V Zhuang X Sreenivasan K Banks S Yang Z Bernick C. Multimodal MRI signatures of cognitive impairment in active professional fighters. Radiology. (2017) 285:555–67. 10.1148/radiol.2017162403 28741982 PMC5673052

[13] Lee B Bennett L Bernick C Banks S. The relationship among depression, cognition and brain volume in professional fighters. Head Trauma Rehabil. (2019) 34:E29–E39. 10.1097/HTR.0000000000000495 31033751

[14] Banks SJ Mayer B Shin W Lowe M Phillips M Modic M. Impulsiveness in professional fighters. J Neuropsychiatry Clin Neurosci. (2014) 26:44–50. 10.1176/appi.neuropsych.12070185 24515676

[15] Stern RA Daneshvar DH Baugh CM Seichepine DR Montenigro PH Riley DO. Clinical presentation of chronic traumatic encephalopathy. Neurology. (2013) 81:1122–9. 10.1212/WNL.0b013e3182a55f7f 23966253 PMC3795597

[16] Bernick C Banks S Phillips M Lowe M Shin W Obuchowski N. Professional fighters brain health study: rationale and methods. Am J Epidemiol. (2013) 178:280–6. 10.1093/aje/kws456 23735309

[17] Gualtieri CT Johnson LG. Reliability validity of a computerized neurocognitive test battery, CNS Vital Signs [Comparative Study Research Support, Non-U.S. Gov't]. Arch Clin Neuropsychol. (2006) 21:623–43. 10.1016/j.acn.2006.05.007 17014981

[18] Simon M Maerlender A Metzger K Decoster L Hollingworth A Valovich McLeod T. Reliability and concurrent validity of select C3 Logix test components. Dev Neuropsychol. (2017) 42:446–59. 10.1080/87565641.2017.1383994 29068702

[19] Kroenke K Spitzer R Williams J. The PDQ - 9: validity of a brief depression severity measure. J Gen Intern Med. (2001) 16:606–13. 10.1046/j.1525-1497.2001.016009606.x 11556941 PMC1495268

[20] Potts G George M Martin L Barratt ES. Reduced punishment sensitivity in neural systems of behavior monitoring impulsive individuals. Neurosci Lett. (2006) 397:130–4. 10.1016/j.neulet.2005.12.003 16378683

[21] McCoy C. Understanding the use of composite endpoints in clinical trials. West J Emerg Med. (2018) 19:947–51. 10.5811/westjem.2018.4.38383 30013696 PMC6040910

[22] Goldberg R Gore J Barton B Gurwitz J. Individual and composite study endpoints: separating the wheat from the chaff. Am J Med. (2014) 127:379–84. 10.1016/j.amjmed.2014.01.011 24486289 PMC4019929

[23] International Conference on Harmonization of Technical Requirements for Registration of Pharmaceuticals for Human Use. ICH harmonized tripartite guideline: statistical principles for clinical trials. Stat Med. (1999) 18:1905–42. 10532877

[24] Bernick C Banks S. What boxing tells us about repetitive head trauma and the brain. Alzheimers Res Ther. (2013) 5:23. 10.1186/alzrt177 23731821 PMC3706825

[25] Montenigro PH Baugh CM Daneshvar DH Mez J Budson AE Au R. Clinical subtypes of chronic traumatic encephalopathy: literature review and proposed research diagnostic criteria for traumatic encephalopathy syndrome. Alzheimers Res Ther. (2014) 6:68. 10.1186/s13195-014-0068-z 25580160 PMC4288217

[26] Food and Drug Administration. Early Alzheimer's Disease: Developing Drugs for Treatment. Guidance for Industry (2018).

[27] European Medicines Agency. Guideline on the Clinical Investigation of Medicines for the Treatment of Alzheimer's Disease (2018).

[28] Weiner M Veitch D Aisen P Beckett LA Cairns NJ Cedarbaum J. 2014 Update of the Alzheimer's disease neuroimaging initiative: a review of papers published since its' inception. Alzheimers Dement. (2015) 11:e1–120. 10.1016/j.jalz.2014.11.001 26073027 PMC5469297

[29] DeKosky ST Blennow K Ikonomovic MD Gandy S. Acute and chronic traumatic encephalopathies: pathogenesis and biomarkers. Nat Rev Neurol. (2013) 9:192–200. 10.1038/nrneurol.2013.36 23558985 PMC4006940

[30] Montenigro P Bernick C Cantu R. Clinical features of repetitive traumatic brain injury and chronic traumatic encephalopathy. Brain Pathol. (2015) 25:304–17. 10.1111/bpa.12250 25904046 PMC8029369

