# Supplementary material for: Assessing Clinical Change in Individuals Exposed to Repetitive Head Impacts: The Repetitive Head Impact Composite Index
Source: Front Neurol. 2021 Jul 6;12:605318. doi: 10.3389/fneur.2021.605318 (PMC8290321; doi:10.3389/fneur.2021.605318)
Supplement: Supplementary file 1 [file Table_1.DOCX]

Table 1-a

p-values for pairwise group comparisons after controlling for the multiple comparisons: Tukey approach for continuous outcomes and Bonferroni approach for categorical outcomes. "NA" represents the cases when the control group has zero events (e.g., the number of fights)

|  | Active Fighters  VS  Retired Fighters | Active Fighters  VS  Control | Retired Fighters  VS  Control |
| --- | --- | --- | --- |
| Age | <0.0001 | <0.0001 | 0.0246 |
| Education Years | 0.9712 | 0.0388 | 0.1707 |
| Female | 0.4839 | 1 | 1 |
| Number of fights | 0.0015 | NA | NA |
| Years of fighting | <0.0001 | NA | NA |
| Race | 0.9954 | 1 | 1 |
